# Chyloperitoneum following robotic-assisted sleeve gastrectomy: a case report

**DOI:** 10.1093/jscr/rjag410

**Published:** 2026-05-31

**Authors:** Jillian Cassidy, Alexander Hanna, William Sebastian, Michelle Abramova, Emily Rienzo, Seth Kipnis

**Affiliations:** Department of Surgery, Jersey Shore University Medical Center Hackensack Meridian Health, 1945 State Route 33, Neptune, NJ 07753, United States; Hackensack Meridian School of Medicine, 123 Metro Blvd, Nutley, NJ 07110, United States; Department of Surgery, Jersey Shore University Medical Center Hackensack Meridian Health, 1945 State Route 33, Neptune, NJ 07753, United States; Department of Surgery, Jersey Shore University Medical Center Hackensack Meridian Health, 1945 State Route 33, Neptune, NJ 07753, United States; Department of Surgery, Jersey Shore University Medical Center Hackensack Meridian Health, 1945 State Route 33, Neptune, NJ 07753, United States; Department of Surgery, Jersey Shore University Medical Center Hackensack Meridian Health, 1945 State Route 33, Neptune, NJ 07753, United States; Hackensack Meridian School of Medicine, 123 Metro Blvd, Nutley, NJ 07110, United States

**Keywords:** lymphatic injury, chyloperitoneum, bariatric surgery, post-operative chylous ascites, gastric sleeve

## Abstract

A 34-year-old female underwent robotic-assisted laparoscopic sleeve gastrectomy and was readmitted with large-volume chylous ascites. Initial non-operative management with aspiration and drainage was attempted but failed, leading to reaccumulation. This necessitated re-operation. Chyloperitoneum after sleeve gastrectomy is rare.

## Introduction

Lymphatic injury resulting in chyloperitoneum is a rare complication of bariatric surgery. Chyloperitoneum may occur by direct leakage through a lymphoperitoneal fistula, through exudate from a retroperitoneal vessel wall, or after rupture of the dilated bowel wall or mesenteric lymphatic vessels [1]. This usually occurs due to obstruction or injury near the mesentery, the cisterna chyli, or the thoracic duct [1]. In laparoscopic sleeve gastrectomy, injury is usually iatrogenic from dissection near the crura. Only five cases of lymphatic injury following sleeve gastrectomy were reported in a systematic review [2]. We present a case of a 34-year-old female with postoperative chyloperitoneum after robotic/laparoscopic sleeve gastrectomy.

## Case report

This is a case of a 34-year-old female with a preoperative body mass index (BMI) of 53 kg/m^2^ who developed persistent nausea, vomiting, and abdominal distension following robotic-assisted sleeve gastrectomy. Consent was obtained for gastric sleeve. The procedure used the Titan SGS™ stapler (Teleflex/Standard Bariatrics, Cincinnati, OH). An intraoperative leak test showed no gastric leak.

**Figure 1:**
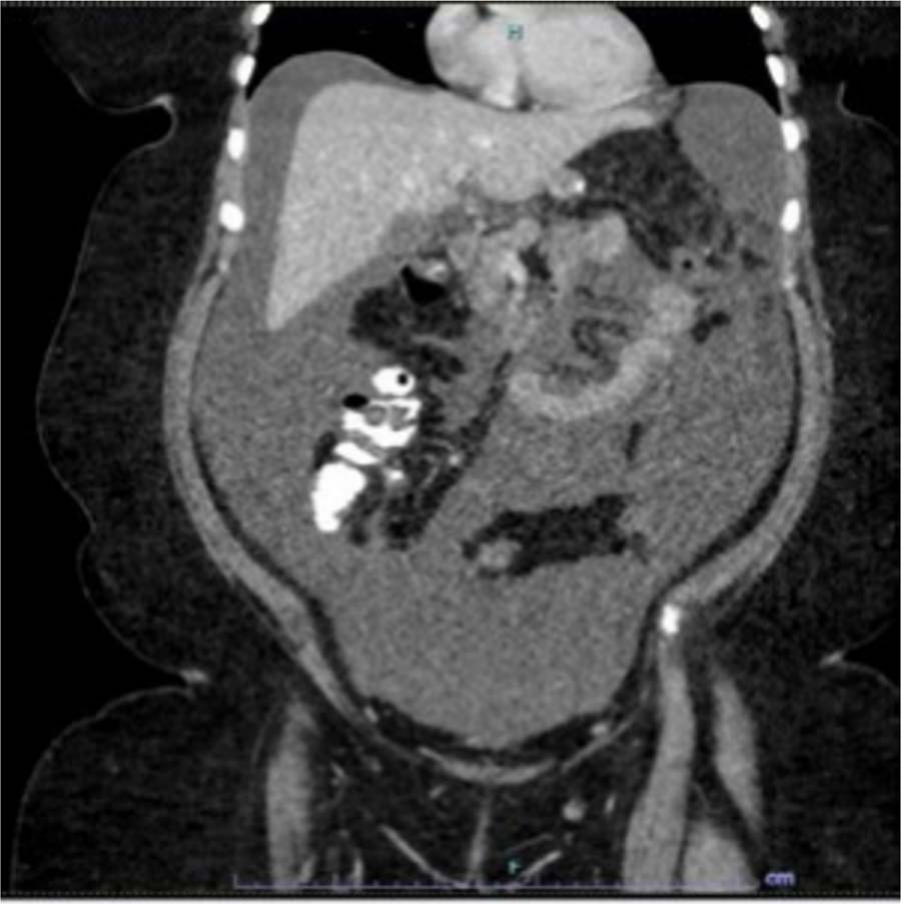
CT scan showing large volume abdominal ascites at post operative day 51.

Following surgery, the patient reported an initial bout of nausea and vomiting that was self-limited. By POD 51, the patient returned again, this time with severe vomiting. Computed tomography (CT) showed a large volume of abdominal ascites without extravasation (Fig. 1). Abdominal ultrasound showed normal portal circulation and no thrombosis.

Paracentesis removed 600 cc of clear, amber fluid. The following day, CT showed reaccumulation of fluid in the peritoneum, and an additional 650 cc of milky ascites was drained. After fluid reaccumulated following paracentesis, a chyle leak was suspected. Chylous appearance, elevated triglycerides, and rapid ascites recurrence supported this suspicion. After discussion with the patient, conservative management was initiated, which included octreotide therapy and a low-fat diet. Lymphoscintigraphy could not be performed due to hospital resource limitations.

**Figure 2:**
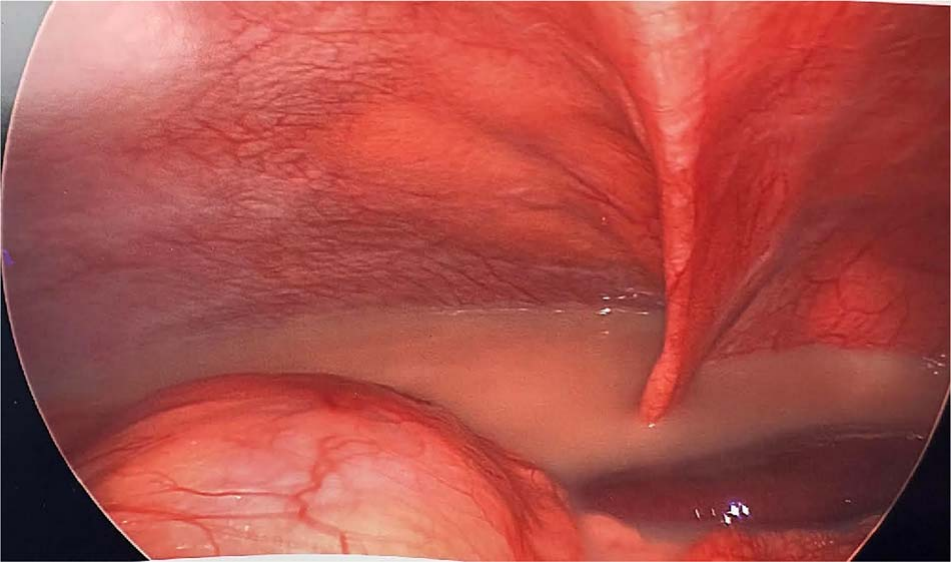
Intraoperative findings consistent with chyloperitoneum.

Persistent chylous ascites prompted re-operation 71 days after initial sleeve gastrectomy. Diagnostic laparoscopy, washout, and drain placement were performed. Six liters of milky ascitic fluid were evacuated (Fig. 2). No sleeve abnormality was found. A prominent, dilated structure on the anterior sleeve surface appeared to be the leak site. Intraoperative identification of a leaking lymphatic trunk indicated an iatrogenic injury near the cisterna chyli. This was likely worsened by the patient’s high preoperative BMI and retroperitoneal lymphatic tension. Laparoscopic clips were placed prophylactically. Two Jackson-Pratt drains were inserted, one subhepatic and one pelvic, for continued drainage. The patient was discharged with drains in place. Over 15 days, the drain output decreased and became clearer. Both drains were then removed. At 3-month routine outpatient follow-up after takeback surgery, no leakage was noted. The patient has remained asymptomatic for 2 years, with no recurrence of chyloperitoneum. Early surgical intervention for high-output lymphatic leaks supports durable outcomes without affecting bariatric success.

## Discussion

Lymphatic injury and chyloperitoneum are uncommon complications in gastric surgery, especially sleeve gastrectomy. Only a few case studies have been reported in the literature on chyloperitoneum after sleeve gastrectomy. Chyloperitoneum is more commonly reported in procedures with dedicated lymph node resection [1].

Symptoms of chyloperitoneum can mimic postoperative symptoms after gastric sleeve surgery: abdominal fullness, bloating, nausea, and vomiting. If these symptoms are severe, persistent, or do not respond to medications and dietary changes, further abdominal imaging should be performed.

The rarity of this complication is highlighted by a 2022 review by Sakran *et al*. They identified only 40 patients undergoing bariatric surgery who developed postoperative chyloperitoneum or chylothorax (38 with chyloperitoneum, 2 with chylothorax). Of these, only 5 underwent sleeve gastrectomy. Most cases followed Roux-en-Y gastric bypasses and were linked to internal hernia, causing lymphatic obstruction at the mesentery root. The cisterna chyli and its tributaries vary anatomically and are often close to the diaphragmatic crura and the posterior aspect of the stomach. In this case, the disruption likely occurred at a major lymphatic branch of the cisterna. This illustrates that, despite advances in robotic precision and stapling technology, the large retroperitoneal lymphatic channels remain at risk during gastric fundus mobilization or dissection near the crus.

Notably, 50%–90% of chyle originates from the intestines and liver [1]. Fasting status before surgery can dramatically decrease lymphatic flow, dropping it to ˂1 ml/min. After a normal diet is resumed, lymphatic flow may rise to over 200 ml/min [1]. This may have contributed to the delayed identification of lymphatic leak after gastric sleeve surgery. Our patients are on a low-calorie, liquid diet for 7 days before and after surgery. As patients add more varied, higher-fat foods, this will increase lymphatic flow and fluid buildup in the abdomen.

Diagnosis involves imaging to identify fluid collections, followed by drainage and analysis. The chylous fluid is composed of lymph and chylomicrons, and the drained fluid will be odorless, milky/clear, sterile, and triglyceride-rich (>200 mg/dL or 2–8-fold the plasma value) [1, 3]. Treatment of lymphatic leak after bariatric surgery typically begins with conservative measures. Often beginning with total parenteral nutrition (TPN), low-fat and high-protein diets, and abdominal drainage. This is an effort to reduce chyle drainage. The addition of octreotide or somatostatin analogs can also help decrease chyle output [4]. For patients who continue to have high drainage output and are refractory to conservative management, consideration of lymphangiography and embolization may be an option, or surgical intervention may be necessary [2]. Lymphoscintigraphy may still be helpful in localizing the source of the leak prior to surgical intervention [1]. In the systematic literature review, ultimately, 18/41 patients required laparoscopic return to the operating room [2]. Although resolution of lymphatic leak and chyloperitoneum after bariatric surgery may require a step-up approach and time to resolution, mortality was not seen as a direct result of lymphatic leak [2].

Chyloperitoneum is an exceedingly rare but important complication following sleeve gastrectomy, with only a few cases reported in the literature. Our patient’s case highlights the diagnostic and management challenges associated with delayed presentation, as symptoms may closely mimic common postoperative complaints. A high index of suspicion is therefore essential, particularly in patients with persistent or progressive symptoms beyond the expected postoperative course.

## References

[1] Lv S, Wang Q, Zhao W et al. A review of postoperative lymphatic leakage. Oncotarget 2017;8:69062–75. 10.18632/oncotarget.17297 28978181 PMC5620321

[2] Sakran N, Parmar C, Ahmed S et al. Chyloperitoneum and chylothorax following bariatric surgery: a systematic review. Obes Surg 2022;32:2764–71. 10.1007/s11695-022-06136-3 35674980

[3] Cleveland Clinic . Chyle: What It Is, Function & Formation. 2025. https://my.clevelandclinic.org/health/body/chyle (27 March 2026, date last accessed).

[4] Pessotti CF, Jatene IB, Buononato PE et al. Use of octreotide in the treatment of chylothorax and chyloperitoneum. Arq Bras Cardiol 2011;96:140–7. 10.1590/S0066-782X2011005000001 22002034

